# Palliative care education in Zagreb – an assessment of the effectiveness of an undergraduate course

**DOI:** 10.3325/cmj.2013.54.212

**Published:** 2013-04

**Authors:** David Oliver, Davor Ježek

**Affiliations:** 1 Consultant in Palliative Medicine, Wisdom Hospice, Rochester, UK; 2 Honorary Reader, University of Kent, Canterbury, UK; 3 Department of Histology and Embryology, Zagreb University School of Medicine, Zagreb, Croatia

Palliative care in Croatia is insufficiently developed. Although the Croatian Society for Hospice and Palliative Care was formed by Professor Anica Jusić in 1989 (1) there have been limited and local developments of services – within certain cities and towns, usually as a result of local enthusiastic doctors or other professionals (2). However there has been growing awareness of the need for education of medical and nursing students and when the English Course for Medical Students was started in Zagreb in 2003 it was agreed that there would be an optional course in palliative care.

Dr David Oliver was elected as Visiting Professor and has been leading the course for five years. He is a Consultant in Palliative Medicine at the Wisdom Hospice in Rochester and holds an honorary post as Reader (Associate Professor) at the University of Kent in the UK. Over the years the course has continued to develop and has included more local Croatian teachers.

The course comprises sessions on the principles of palliative care, the basic principles of pain and symptom management, psychosocial care, communication, family care, and bereavement. It is held over three days and there is a short examination at the end. The aim has been to have an interactive approach, using audio-visual presentations and case discussions, as well as more didactic lectures. It is held at the end of the third year, when students have only just started to experience patient involvement.

Every year the course has been evaluated and the students have been asked to complete a short questionnaire on their understanding and attitudes to end-of-life care, based on the previous Self-efficacy in Palliative Care Scale and parts of the Thanatophobia Scale (3). In 2012, all sessions were evaluated at better than 4 on a 0 to 5 point scale and the overall usefulness of the course was evaluated at 4.4 out of 5.

Results from 2008- 2012 showed evidence of increasing confidence in all areas – talking about dying, discussing cancer, assessing symptoms, discussing psychological issues, and working as part of a multidisciplinary team – with improved scores (Table 1). There also seems to be a movement to feeling less uneasy when coping with dying patients but little change is observed in discussing dying and death, which again may be related to their limited patient exposure.

**Table 1:** Results of questionnaire on attitudes and feelings

	Start of module (n = 72)	End of module (n = 73)
Discussion of death and dying*	65	74
Discussion of effect of cancer with patients*	62	72
Assessment of symptoms of cancer patient*	66	74
Discussion and provision of psychological care*	52	74
Working in multidisciplinary team*	71	80
Dying patients make me feel uneasy^†^	3.6	3.2
When patients begin to discuss death, I feel uncomfortable^†^	3.0	3.2

*0 = very anxious, 100 = very confident.

†Score of feelings (1 = strongly disagree, 7 = strongly agree).

These results would support the effectiveness of education in palliative care, and it would seem that, even at this early stage in their training, students can gain more confidence and change their attitudes to dying patients. Within Zagreb this has led to the development of the Centre for Palliative Medicine, Medical Ethics and Communication Skills (4) and communication skills are now to be taught within every year of the curriculum.

Palliative care is an essential part of the care that is offered to patients and their families and we hope that this small contribution will encourage other medical and nursing schools to introduce similar courses. Within the English Course in Zagreb it is hoped that the education can be extended to all students within the Medical Faculty and both at this early stage and later in the curriculum, when they will have gained more confidence in the care of patients and their families.
